# Making the case for an International Decade of Radiocarbon

**DOI:** 10.1098/rsta.2023.0081

**Published:** 2023-11-27

**Authors:** Timothy I. Eglinton, Heather D. Graven, Peter A. Raymond, Susan E. Trumbore, Lihini Aluwihare, Edouard Bard, Sourish Basu, Pierre Friedlingstein, Samuel Hammer, Joanna Lester, Jonathan Sanderman, Edward A. G. Schuur, Carlos A. Sierra, Hans-Arno Synal, Jocelyn C. Turnbull, Lukas Wacker

**Affiliations:** ^1^ Department of Earth Sciences, ETH Zurich, Zurich, Switzerland; ^2^ Department of Physics, ETH Zurich, Zurich, Switzerland; ^3^ Department of Physics, Imperial College London, London, UK; ^4^ School of the Environment, Yale University, New Haven, CT, USA; ^5^ Department of Biogeochemical Processes, Max Planck Institute for Biogeochemistry, Jena, Germany; ^6^ Geosciences Research Division, Scripps Institution of Oceanography, University of California, San Diego, La Jolla, CA, USA; ^7^ CEREGE, Aix-Marseille University, CNRS, IRD, INRAE, Collège de France, Aix-en-Provence, France; ^8^ Global Modeling and Assimilation Office, NASA Goddard Space Flight Center, Greenbelt, MD, USA; ^9^ Earth System Science Interdisciplinary Center, University of Maryland, College Park, MD, USA; ^10^ College of Engineering, Mathematics, and Physical Sciences, University of Exeter, Exeter, UK; ^11^ Institut für Umweltphysik, Heidelberg University, Heidelberg, Germany; ^12^ Woodwell Climate Research Center, Falmouth, MA, USA; ^13^ Center for Ecosystem Science and Society, Northern Arizona University, Flagstaff, AZ, USA; ^14^ Rafter Radiocarbon Laboratory, GNS Science, Lower Hutt, New Zealand; ^15^ CIRES, University of Colorado at Boulder, Boulder, CO, USA

**Keywords:** radiocarbon (^14^C), carbon cycle, climate change, Anthropocene, fossil fuels, bomb ^14^C

## Abstract

Radiocarbon (^14^C) is a critical tool for understanding the global carbon cycle. During the Anthropocene, two new processes influenced ^14^C in atmospheric, land and ocean carbon reservoirs. First, ^14^C-free carbon derived from fossil fuel burning has diluted ^14^C, at rates that have accelerated with time. Second, ‘bomb’ ^14^C produced by atmospheric nuclear weapon tests in the mid-twentieth century provided a global isotope tracer that is used to constrain rates of air–sea gas exchange, carbon turnover, large-scale atmospheric and ocean transport, and other key C cycle processes. As we write, the ^14^C/^12^C ratio of atmospheric CO_2_ is dropping below pre-industrial levels, and the rate of decline in the future will depend on global fossil fuel use and net exchange of bomb ^14^C between the atmosphere, ocean and land. This milestone coincides with a rapid increase in ^14^C measurement capacity worldwide. Leveraging future ^14^C measurements to understand processes and test models requires coordinated international effort—a ‘decade of radiocarbon’ with multiple goals: (i) filling observational gaps using archives, (ii) building and sustaining observation networks to increase measurement density across carbon reservoirs, (iii) developing databases, synthesis and modelling tools and (iv) establishing metrics for identifying and verifying changes in carbon sources and sinks.

This article is part of the Theo Murphy meeting issue 'Radiocarbon in the Anthropocene'.

## 1. Importance of radiocarbon in studying the global C cycle

^14^C, the radioactive isotope of carbon (half-life *ca* 5700 years), produced naturally in the atmosphere by cosmic rays and rapidly incorporated into CO_2_, undergoes radioactive decay as it is distributed between the land, ocean and atmospheric reservoirs. Tracing this distribution provides insights into carbon cycle processes that occur on a range of timescales [1,–3]. On multi-century to millennial timescales associated with deep ocean circulation, sediment burial or soil formation, radioactive decay of ^14^C provides a measure of how long carbon has been isolated from exchange with the atmosphere (e.g. [4,5]). Past changes in atmospheric ^14^C production, and distribution of ^14^C among ocean and land C reservoirs are recorded in the atmospheric record preserved in tree rings (e.g. [6,–8]) and marine archives [9]. Such variations have provided the basis for extensive application of ^14^C as a dating tool, and in paleoclimate and paleoenvironmental reconstructions [10]. Recent human alteration of ^14^C has provided additional ways to trace C cycling on timescales of years to decades.

**Figure 1.**
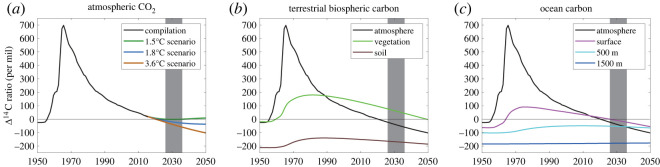
Radiocarbon in the Anthropocene. (*a*) Measured Δ^14^C (^14^C/C) in atmospheric CO_2_ between 1950 and 2015 from a compilation [12] together with future projections for the global mean Δ^14^C in three scenarios with different greenhouse gas emissions trajectories and different global average temperatures in 2100 (1.5°C: SSP1–1.9, 1.8°C: SSP1–2.6 and 3.6°C: SSP3–7.0; [3]). The compilation combines various data sources and is representative of tropical regions [12]. Data are reported with typical normalization and correction [13]. (*b*) Simulated global mean Δ^14^C in terrestrial vegetation and soil carbon and (*c*) simulated global mean Δ^14^C in ocean carbon at the surface and at 500 m and 1500 m depths from the CESM2 model's historical and SSP3–7.0 experiments of the Large Ensemble (member 1001.001) following CMIP6 protocols [14,15]. Also plotted in (*b*) and (*c*) is the Δ^14^C in atmospheric CO_2_ from the compilation and the SSP3-7.0 scenario. In (*b*) areas poleward of 60° are excluded. The grey vertical bars indicate the proposed International Decade of Radiocarbon.

The burning of carbon in fossil fuels that was fixed millions of years ago and thus has no remaining ^14^C dilutes the isotopic ratio of ^14^C/^12^C in atmospheric CO_2_. Suess [11] first measured this decline and used it to link an observed rise in CO_2_ with the burning of fossil fuels. As fossil fuel emissions have increased since the industrial revolution, so has this ‘Suess effect’ (figure 1).

Variations in atmospheric ^14^C due to natural and fossil effects were dwarfed by the production of ^14^C during atmospheric nuclear weapons testing in the 1950s and early 1960s (figure 1). This ‘bomb’ ^14^C, most of which was produced in a few large nuclear tests just before the Partial Nuclear Test Ban treaty went into effect in 1963, nearly doubled atmospheric ^14^C/^12^C in the Northern Hemisphere in a few years, producing a global isotope tracer that has since propagated into ocean and land C reservoirs.

Over the decades since 1963, the ^14^C/^12^C of the atmosphere declined as bomb ^14^C mixed into terrestrial and oceanic carbon reservoirs, and by ongoing dilution by fossil C that is playing an increasingly important role. The level of ^14^C/^12^C in atmospheric CO_2_ has now dropped back to its preindustrial level [16]. This decreasing trend will persist if fossil fuel emissions continue, but it will cease if fossil fuel emissions are curtailed ([3,17]; figure 1). We are now in a new era in which gradients are reversed: ^14^C/^12^C of atmospheric CO_2_ becomes lower than that in the surface ocean and land biosphere [16,17]. This important juncture highlights the unique sensitivity of ^14^C to changes in the balance of processes modulating Earth's carbon cycle, and also highlights the value of time-series observations of important reservoirs to track trajectories of future change.

Despite longstanding recognition of the diagnostic power of ^14^C as both a tracer and in-built ‘clock’ for understanding carbon cycle processes and carbon cycle change, its potential is far from fully realized. This is largely due to the logistical challenges and costs that have traditionally been associated with preparing and analysing samples for ^14^C content. As a consequence, ^14^C measurements have been applied sparingly, largely in support of other analyses. Especially during the early decades following the 1960s, radiocarbon laboratories used decay-counting methods that required large (greater than 1 g C) samples and the global capacity was of the order of hundreds of samples per year in a given laboratory. The advent of accelerator mass spectrometry (AMS) in the 1980s greatly alleviated sample size requirements, but initially few instruments were in operation. Moreover, ^14^C measurements have largely been contained within specific disciplines or within expert groups, resulting in a lack of freely accessible datasets and modelling tools. Although ^14^C is recommended to be included in international observation programmes (https://gcos.wmo.int/en/essential-climate-variables/; [18]) as well as in earth system modelling [19,20], only one model incorporating ^14^C in both land and ocean components has reported output in the latest version of the carbon-climate model intercomparison effort CMIP6 (CESM2, https://esgf-node.llnl.gov/projects/cmip6/; [14,21]). There has also been a perception in some research communities that the utility of ^14^C has diminished as atmospheric ^14^C/^12^C has dropped over the decades since the bomb tests. These considerations have resulted in limited usage of radiocarbon by the carbon cycle community despite the potential it holds for understanding key processes. In particular, ^14^C can provide vital constraints for processes that influence C cycling on decadal to centennial timescales that are needed to understand current and future anthropogenic carbon uptake and storage, including deliberate carbon removal activities (e.g. [22]).

## 2. Current opportunities

Recent years have seen dramatic advances in ^14^C measurement speed, versatility and sensitivity; a trend that will likely continue. Moreover, the advent of compact, lower-cost AMS systems [23], including those with CO_2_-accepting ion sources [24], has resulted in a rapid increase in the number of AMS instruments installed in different laboratories worldwide and an associated increase in global capacity that is likely now well in excess of 100 000 ^14^C measurements per year. This change allows for contemplation of ^14^C measurement programmes that would have been unthinkable a few decades ago in terms of scope, sample type and size. For the study of specific processes, the increased sensitivity of AMS has even allowed for new applications of ^14^C as a deliberately added low-level tracer (e.g. [25]).

Concomitant with these instrumental advances is the implementation of ^14^C into new modelling tools, including next-generation earth system models of the global carbon cycle [14] and atmospheric inverse models for regional GHG source identification [26]. New methods are now available for offline simulation of ^14^C from the numerical output of carbon-climate models [27]. Furthermore, new databases have been initiated and are being actively updated to compile previously disparate observations (e.g. [28–30]).

Given these developments, we see many powerful ^14^C applications that could start or expand with more coordinated action to observe, compile and interpret ^14^C data. Specific initiatives include: (i) verification/attribution of changes in fossil fuel emissions of CO_2_, CH_4_ and aerosol carbon. For example, measurements of ^14^C/^12^C have enabled the evaluation of officially reported fossil fuel CO_2_ emissions [12,26] including local fossil-derived enhancements in CO_2_ [30–32], while ^14^C in aerosol smoke helps identify the age of burned C [34] or the fossil fraction of anthropogenic aerosol sources [35]; (ii) elucidation of processes and timescales involved in carbon storage by tracing bomb ^14^C incorporation over time. Mitigation strategies for sequestering C, for example through land management, must also assess how long C will remain in storage [36,37]; (iii) detection and attribution of C loss from reservoirs most vulnerable to change. These include release of older C from thawing permafrost [38,39] or changing ventilation of various parts of the global ocean [40,41]; (iv) testing of the basic understanding built into carbon cycle models at a range of scales, either through use of models to predict ^14^C values for comparison with observations or to infer transit time and age distributions within carbon pools (e.g., soils); (v) application of deliberate tracer approaches to determine reaction rates or follow the partitioning of carbon over many months and years. For example, an experiment with ^14^C enrichment in a whole forest provided strong evidence that C in forest soils is more derived from roots than from surface litter [42].

## 3. The need for a ‘Decade of Radiocarbon’

Maximizing the benefits of the changing radiocarbon tracer, especially given the current switch in gradients between atmosphere, ocean and land, will require a coordinated observational and modelling effort over a sustained period: an ‘International Decade of Radiocarbon’. Similar to the International Polar Years in 1882–1883, 1932–1933, 1957–1958 and 2007–2008 (next planned for 2032–2033), this would involve a comprehensive global census to quantify the present and past distributions of ^14^C across Earth's dynamic carbon pools over the past decades that can guide sampling into the future (box 1). We propose a decade-long programme rather than only 1 or 2 years since some of the activities will need more time to implement or require multiple stages. Along with new observations, a concerted effort to compile existing and emerging data, and to develop and share teaching and modelling tools, will be essential to expand the use and utility of ^14^C in carbon cycle studies. This initiative requires cooperation among scientists, funders and other parties across many countries, particularly those which are underrepresented with respect to existing observations.

Box 1. **A decade of radiocarbon—specific needs.**

*Measurements*

— Geographical expansion of records of radiocarbon in air in under-sampled areas including over the Southern Ocean and the tropics and in continental and urban areas for verification of changes in fossil fuel emissions at local/regional scales.
— Identification, measurement and preservation of archived samples and natural archives (e.g. soils, sediments, speleothems, corals, tree rings, waters) that can strategically add to tracing bomb ^14^C through carbon pools over the last decades.
— New measurements to document ongoing changes in ^14^C in C reservoirs, particularly those involving losses of older, yet vulnerable carbon pools to climate change (e.g. via increased decomposition of rapidly warming high latitude soils).
— Development of new ^14^C tracer labelling experiments to determine carbon uptake rates and turnover times.

*Synthesis*

— Data discovery to recover and make historical data accessible.
— Expansion of existing and creation of new databases that collate older data and provide repositories for new data (with key metadata) for specific carbon reservoirs, including the atmosphere, soils, ocean waters and sediments in a coordinated fashion.
— Generation of data products for use as common benchmarks for models.
— Linkage to data quality and intercomparison efforts such as the Greenhouse Gas Measurement Techniques [43] and Radiocarbon intercomparisons (e.g. [44]) to ensure ongoing data quality improvements.
— Inter-laboratory comparison of ^14^C measurements on the same samples in order to validate data, establish across-laboratory calibrations, and facilitate data homogenization and dissemination [44,45].

*Modelling*

— Development of best practice recommendations and common tools for ^14^C modelling in various realms, including off-line simulations based on existing carbon cycle models.
— Development of tools and approaches for integrating radiocarbon processes in carbon cycle models and evaluation against observations.
— Engagement with the Earth System Modelling community and other communities to promote use of ^14^C observations into model developments for process understanding and evaluation.

In some cases, radiocarbon analyses could be added to ongoing efforts to characterize C stocks and fluxes. However, this ‘add-on’ approach is how ^14^C measurements have been made in the past, and has led to the current uneven patchwork of measurements that can be difficult to synthesize. Global measurements of ^14^C/^12^C in atmospheric CO_2_, critical to applications across a variety of fields, have been largely maintained by individual researchers [26,46–49] via insecure and intermittent funding. Samples are sometimes stored for long periods before analysis, introducing latency global atmospheric records. Spatial coverage has been sparse and sporadic, particularly for key sites in the tropics and the Southern Hemisphere. Huge efforts went into surveying ^14^C in the oceans during coordinated ocean sampling campaigns like GEOSECS, WOCE and GLODAP [50] and into ongoing repeat transects in GO-SHIP. However, measurements of ^14^C in oceanic dissolved inorganic carbon are no longer a level-1 priority and presently only one or two cruises per year are sampled for ^14^C measurements, despite their continued value for assessing ocean ventilation and circulation [51]. Measurements of ^14^C/^12^C in soils are often only available for one point in time even though repeated measurements can provide much stronger constraints on carbon turnover [52], and they are overwhelmingly concentrated in temperate forest biomes. Radiocarbon measurements of C fluxes—for example respiration from soils or plants—also provide direct constraints on how fast C transits complex systems [53] but are even sparser.

Given both the burgeoning global capacity to measure ^14^C and number of researchers who use this important tool, as well as the urgency to make the most of inadvertent global tracer experiments resulting from fossil fuel use and bomb testing, we find it time to bring the geophysical and biogeochemical communities together to forge an International Decade of Radiocarbon measurement, integration and modelling. It would be a great shame if we miss the opportunity to harness the power of ^14^C as a tool to understand our changing carbon cycle, verify changes in fossil emissions, and evaluate the efficacy of nature-based carbon removal practices currently being developed [54].

To realize the vision of the International Decade of Radiocarbon beginning in the late 2020s we have started to promote discussions at relevant international conferences including the Radiocarbon in the Anthropocene meeting at Whittlebury Park, UK (May 2022), and at the 24^th^ Radiocarbon conference in Zurich, Switzerland (September 2022). The purpose of this Opinion Piece is to further highlight this initiative and to catalyse future discussions, coordination and planning activities.

## Data Availability

CMIP6 output is available at https://esgf-node.llnl.gov/search/cmip6/.
